# An animal derivative-free medium enhances *Lactobacillus johnsonii *LJO02 supernatant selective efficacy against the methicillin (oxacillin)-resistant *Staphylococcus aureus *virulence through key-metabolites

**DOI:** 10.1038/s41598-022-12718-z

**Published:** 2022-05-23

**Authors:** Diletta Francesca Squarzanti, Paola Zanetta, Margherita Ormelli, Marcello Manfredi, Elettra Barberis, Virginia Vita Vanella, Angela Amoruso, Marco Pane, Barbara Azzimonti

**Affiliations:** 1 Laboratory of Applied Microbiology, Department of Health Sciences (DiSS), Center for Translational Research on Allergic and Autoimmune Diseases (CAAD), School of Medicine, Università del Piemonte Orientale (UPO), Corso Trieste 15/A, 28100 Novara, Italy; 2 Laboratory of Biological Mass Spectrometry, Department of Translational Medicine (DiMeT), Center for Translational Research On Allergic and Autoimmune Diseases (CAAD), School of Medicine, Università del Piemonte Orientale (UPO), Corso Trieste 15/A, 28100 Novara, Italy; 3 Probiotical Research S.R.L, Via Mattei 3, 28100 Novara, Italy

**Keywords:** Microbiology, Medical research

## Abstract

The spread of multidrug-resistant bacteria, such as the skin commensal *Staphylococcus aureus*, is a worldwide health challenge; new methods to counteract opportunistic pathogen growth and virulence are urgent. We compared the activity of *Lacticaseibacillus rhamnosus* LR06 (DSM 21981) and *Lactobacillus johnsonii* LJO02 (DSM 33828) cell-free supernatants (CFSs) produced in the conventional animal derivative-based MRS medium and an innovative animal derivative-free broth (TIL) versus the MDR *S. aureus* (ATCC 43300). CFS influence was assessed towards the viability, metabolic activity, and ability to form biofilm of the MDR strain through optical density, alamarBlue assay, and crystal violet staining; their content in short-chain fatty acids, lactic acid, and proteins was analysed via high-resolution mass spectrometry and gas chromatography. All CFSs reduce viable and metabolically active *S. aureus*, being TIL more efficient compared to MRS in stimulating lactic acid bacteria metabolism and decreasing *S. aureus* biofilm formation. Particularly, the CFS from LJO02 grown in TIL has the best efficacy, revealing a high amount of lactic acid and 59 peculiar proteins; its effectiveness is partially maintained upon trypsin and proteinase K treatments, but not by pepsin and pH basification. Therefore, antagonistic CFSs may represent a strategic prevention approach, with bacteriotherapeutic and bio-repair potential.

## Introduction

*Staphylococcus aureus,* a human skin commensal, can be responsible for several opportunistic endogenous and exogenous infections in different body fluids and tissue districts. Indeed, it can lead to several inflammatory/necrotic disorders like atopic dermatitis, psoriasis, endocarditis, osteomyelitis, and pneumonia. It also contributes to the onset and progression of skin tumors, with serious consequences on human health, life quality, and expectancy^1–8^.

The misuse and overuse of antibiotics (mostly broad-spectrum) in humans and veterinary farms, hygiene products, and food favor *S. aureus* opportunistic pathogen selection, dominance, and natural propensity for multidrug resistance (MDR), causing health care costs to rise^9–11^. Indeed, *S. aureus* belongs to the ESKAPE pathogens (*Enterococcus faecium*, *Staphylococcus aureus*, *Klebsiella pneumoniae*, *Acinetobacter baumannii*, *Pseudomonas aeruginosa,* and *Enterobacter* species), which exhibit intrinsic virulence potential and MDR.

According to the United States Center for Disease Control and Prevention (US CDC), methicillin (oxacillin)-resistant *S. aureus* strains (MRSA) are increasingly responsible for invasive life-threatening hospital-acquired infections with high mortality rates; they spread more and more frequently in the community, also posing a threat to the health of non-hospitalized people^12–15^.

Therefore, it is urgent to study novel strategies, that go beyond antibiotic treatments, capable to revert or at least slow down the fast aggravation of the MDR pathogen expansion^16^. Among the prevention measures, probiotics demonstrate a positive action on human hosts in terms of decreased infection risk, antibiotic need, disease severity, and duration, especially those belonging to the *Lactobacillus* and *Bifidobacterium* genera^17,18^.

Recently, cell-free supernatants (CFSs) from lactic acid bacteria (LAB) displayed the ability to mitigate the virulence of different pathogenic species, among which that of *S. aureus*^19,20^.

On this premise, we examined the selective capability of *Lacticaseibacillus rhamnosus* LR06 and *Lactobacillus johnsonii* LJO02 CFSs, both produced in a medium with (de Man, Rogosa and Sharpe, MRS) or without animal derivatives (TIL), to attenuate the growth and virulence of a MRSA strain. We first assessed culture media pH influence on *S. aureus* growth and virulence. We evaluated which one of the two media was more efficient in stimulating the LAB metabolism, and therefore the capacity of their CFSs in inhibiting the pathogen viability and biofilm formation. The CFS from LJO02 cultured in TIL revealed to be the most efficient among all the tested conditions, so we focused on it. To establish if the pH itself could affect its antibacterial properties, we bring the pH to the same value as the pristine LB medium; to gain more information about its proteic content activity, we treated it with trypsin, proteinase K, or pepsin. Finally, we carried out a short-chain fatty acids (SCFAs), lactic acid, and proteomic qualitative and quantitative analysis of both CFS types, and the media before bacteria inoculation, using a high-resolution mass spectrometry and gas chromatography, to determine a subset of molecules implicated in this phenomenon.

For the first time in our knowledge, we demonstrated that LJO02 adapts to the animal derivative-free TIL medium, and produces high amounts of lactic acid and 59 peculiar metabolites able to limit *S. aureus* growth and biofilm formation.

## Methods

### Bacterial culture conditions and growth curves

*S. aureus* (American Type Culture Collection, ATCC 43300, distributed by LGC Standards S.r.l., Sesto San Giovanni, Milan, Italy) was cultivated overnight (ON) at 37 °C with shaking at 200 revolutions per minute (rpm) in Luria–Bertani broth (LB, Sigma-Aldrich, St. Louis, MO, distributed by Merck Life Science S.r.l., Milan, Italy). The probiotic strains *L. rhamnosus* LR06 and *L. johnsonii* LJO02 (DSM 21981 and DSM 33828, respectively; both from Probiotical Research S.r.l., Novara, Italy) were grown ON at 37 °C in static conditions in (i) MRS broth (Condalab, distributed by Biosigma, Cona, Venice, Italy; formula in g/L: bacteriological peptone 10, dextrose 20, dipotassium phosphate 2, magnesium sulfate 0.2, manganese sulfate 0.05, beef extract 8, sodium acetate 5, tween 80 1, yeast extract 4, ammonium citrate 2), a medium with animal-derived ingredients, specific for the isolation and growth of LAB, and (ii) TIL broth, an animal derivative-free medium containing peptones from plant sources and supplemented with glucose (Probiotical Research S.r.l., Novara, Italy; formula in g/L: proteose peptone N-3 10, dextrose 20, dipotassium phosphate 2, magnesium sulfate 0.1, manganese sulfate 0.05, vegetal extract—confidential-, sodium acetate 5, tween 80 1, yeast extract 5, ammonium citrate 2). Growth curves were assessed for pathogenic bacteria and probiotics in all the above-described media by optical density reading at 600 nm (OD_600_) using the NanoPhotometer NP80 (Implen, Munich, Germany). All the strains were renewed in the proper medium before each experiment.

### Cell-free supernatant production

CFSs were prepared as described by Jeffrey and collaborators in 2020, with few modifications^21^. Briefly, LR06 and LJO02 strains were cultured in MRS and TIL broths for 8 h (h), inoculated at the OD_600_ = 0.05 from an active culture, and then centrifuged at 3000 × g for 20 min (min) at 4 °C (Heraeus Megafuge 16R, Thermo Fisher Scientific, Rodano, Milan, Italy). The supernatants were collected, sterilized with 0.22 μm PES filters (Clearline, distributed by Biosigma), aliquoted, and stored at − 20 °C.

### Culture medium and CFS pH measure

The basal pH of LB, MRS, and TIL media was measured before bacteria inoculation. The pH of CFSs produced by both LAB in MRS and TIL, alone or after being added to LB, was also evaluated (Sension + PH3, Hach Lange S.r.l., Milan, Italy).

### pH and CFS influence assessment

LAB are capable to convert carbohydrate substrates into organic acids (mainly lactic acid) during fermentation, causing culture media acidification. To exclude that medium pH values themselves could influence *S. aureus* viability and capacity to form biofilm during the co-incubation with CFSs, the pathogen was cultured not only in LB but also in the same medium with the addition of an equal volume of LB, MRS, and TIL broths acidified to pH = 4.3 with the strong hydrochloric acid or with the weak lactic acid (Sigma-Aldrich); growth curves were compared to that one in the standard condition (LB medium).

Furthermore, 200 µL of *S. aureus* (initial OD_600_ = 0.035, corresponding to 3.5 × 10^7^ cells/mL) were co-seeded in 48 well-plates in LB with three different CFS types (50% v/v) produced in MRS and TIL: the first two derived from each single probiotic strain, while the last one from the combination of the two probiotic supernatants (MIX condition). *S. aureus* in LB was considered as growth control. To highlight the possible influence of the probiotic media, two further controls were produced with the pathogen in LB medium plus MRS or TIL. *S. aureus* OD_600_ was read in 48 multi-well plates using a Spark microplate reader (Tecan Trading AG, Switzerland) at time 0 (T_0_) and after 24, 48, and 72 h of incubation at 37 °C. For each condition, T_0_ was considered as 100% viability for planktonic cells and biofilm together.

To better assess whether the pH itself could influence the anti-bacterial properties of the CFS from LJO02 in TIL, we basified it with NaOH to pH values of 6.6 (the same as pristine LB).

To achieve information about the activity of the proteins in the CFS from LJO02 in TIL, we treated it with proteolytic enzymes, as described by Tao and colleagues^22^. The CFS, prepared as above described, was treated with trypsin (Corning, distributed by Biosigma), proteinase K (Euroclone S.p.A., Pero, Milan, Italy), or pepsin (Sigma-Aldrich). Briefly, before trypsin and proteinase K treatments, the pH of the CFS was adjusted at 8 using NaOH 5 N to allow the enzymes to work; then the CFS was treated for 90 min at 37 °C either with trypsin or proteinase K at the final concentration of 50 μg/mL. The pH was then readjusted at the value of 4 with HCl. Conversely, the treatment with pepsin (50 μg/mL) was directly performed for 90 min at room temperature, since this enzyme works at acidic pH. All the CFSs were then filtered with 10 kDa cut-off spin columns (Amicon Ultra-0.5 Centrifugal Filter Unit, Millipore, distributed by Sigma Aldrich) to remove the enzymes (molecular weights in kDa: trypsin 24, proteinase K 28.9, and pepsin 34.5, respectively). *S. aureus* cells were co-seeded as above described with the modified CFSs.

All experiments were replicated three times on separate days.

### Planktonic cell and biofilm viability assay

*S. aureus* planktonic cell and biofilm growth was evaluated in each one of the above-described treatment conditions. The planktonic cells were separated from the biofilm and the metabolic activity of both forms was analyzed independently using the alamarBlue assay (resazurin sodium salt, final concentration 0.015%; Sigma-Aldrich). The plates were incubated protected from light for 2 h at 37 °C with the chromogenic substrate, as described by the manufacturer. One hundred µL of each replicate were transferred into a black 96-well plate (Greiner, Sigma-Aldrich); the fluorescence was detected with a Spark microplate reader (ex/em = 535/590 nm). All experiments were replicated three times on separate days.

### Biofilm staining with crystal violet (CV)

To assess the extent of biofilm formation, the protocol described by Stepanovic and collaborators was used, with few modifications^23^. Briefly, at the defined time points, the CFS-treated pathogen and untreated control samples were washed twice with saline solution and fixed with pure methanol for 15 min. Then, 150 μL/well of CV solution (Sigma-Aldrich) were added for 5 min to stain the dried biofilms. The excess amount of CV was removed by washing carefully with tap water. The images were acquired with an EVOS FLoid Imaging System (Thermo Fisher Scientific, Waltham, MA). 3D biofilm pictures were obtained using the ImageJ software (Rasband, W.S., ImageJ, U. S. National Institutes of Health, Bethesda, Maryland, USA, https://imagej.nih.gov/ij/, 1997–2018). Then, CV was dissolved with 33% acetic acid and quantified by measuring the absorbance at 570 nm using a Spark microplate reader. All experiments were replicated three times on separate days.

### Short-chain fatty acids and lactic acid analysis

Basal culture media and CFSs were assessed for SCFAs content after a liquid–liquid extraction method with methyl tert-butyl ether (MTBE). SCFAs were then analyzed using a gas chromatography-mass spectrometer GC-TOFMS (BT, Leco Corp., St. Josef, MI, USA), as previously described^24^. Briefly, the column adopted was a 30 m DB-FATWAX-UI (Agilent Technologies, Santa Clara, CA), while high-purity helium (99,9999%) was used as the carrier gas. One μL of each sample was injected in splitless mode at 250 °C. The program was as follows: the initial temperature was 40 °C for 2 min, then ramped 7 °C/min up to 165 °C, 25 °C/min up to 240 °C, and maintained for 5 min. The electron impact ionization was applied at 70 eV. The ion source temperature was set at 250 °C, the mass range at 40 to 300 m/z with an extraction frequency of 32 kHz and an acquisition rate of 200 spectra/s.

The lactic acid content was measured by using the Megazyme Lactic Acid Assay Kit (NEOGEN Europe Ltd), following the manufacturer’s instructions.

### Proteomic analysis

#### Sample preparation

The proteins in the CFSs were precipitated ON at − 20 °C with 4 volumes of ice-cold acetone. The pellets were then collected by centrifugation at 17,000 × g for 20 min at 4 °C and then resuspended in 100 mM ammonium bicarbonate (NH_4_HCO_3_). Protein concentration was determined using the BCA protein assay (Sigma-Aldrich). Proteins were reduced with DTT 200 mM, subjected to alkylation with iodoacetamide (IAM) 200 mM, and then completely digested with 2 μg of trypsin. The peptide digests were desalted on the Discovery^®^ DSC-18 solid-phase extraction (SPE) 96-well plate (25 mg/well; Sigma-Aldrich)^25^.

#### Proteomic analysis and data processing

The digested peptides were analyzed with a UHPLC Vanquish system (Thermo Scientific, Rodano, Italy) coupled with an Orbitrap Q-Exactive Plus (Thermo Scientific). Peptides were separated by a reverse-phase column (Accucore™ RP-MS 100 × 2.1 mm, particle size 2.6 µm). Mobile A and B phases were water and acetonitrile respectively, both acidified with 0.1% formic acid. The analysis was performed using the following gradient: 0–5 min from 2 to 5% B; 5–55 min from 5 to 30% B; 55–61 from 30 to 90% B and hold for 1 min, at 62.1 min; the percentage of B was set to the initial condition of the run at 2% and hold for about 8 min. The mass spectrometry analysis was performed in positive ion mode with a voltage of 2.8 kV. For the spectra acquisition, a data-dependent (ddMS2) top 10 scan mode was used. Survey full-scan MS spectra (mass range m/z 381 to 1581) were acquired with resolution R = 70,000 and AGC target 3 × 10^6^. MS/MS fragmentation was performed using high-energy c-trap dissociation (HCD) with resolution R = 35,000 and AGC target 1 × 10^6^. The normalized collision energy (NCE) was set to 30.

The mass spectra analysis was carried out using Mascot v. 2.4 (Matrix Science Inc., Boston, USA); the digestion enzyme selected was trypsin, with 2 missed cleavages, a search tolerance of 10 ppm was specified for the peptide mass tolerance, and 0.1 Da for the MS/MS tolerance. The following modifications were specified for the analysis: carbamidomethyl cysteines and oxidized methionine as fixed and variable modifications, respectively^26^. Mass spectra were searched against the NCBI *L. rhamnosus* and *L. johnsonii* sequence databases (2021).

Bioinformatic analysis was performed using the web-based DAVID tool (https://david.ncifcrf.gov).

### Statistical analysis

Unpaired t-test and two-way ANOVA followed by Tukey multiple comparisons were performed using the GraphPad Prism version 6.01 for Windows (GraphPad Software, San Diego, California USA, www.graphpad.com). Results were expressed as mean ± standard deviation (SD). Statistical significance was fixed at *p* < 0.05.

## Results

### Bacterial growth curves

**Figure 1:**
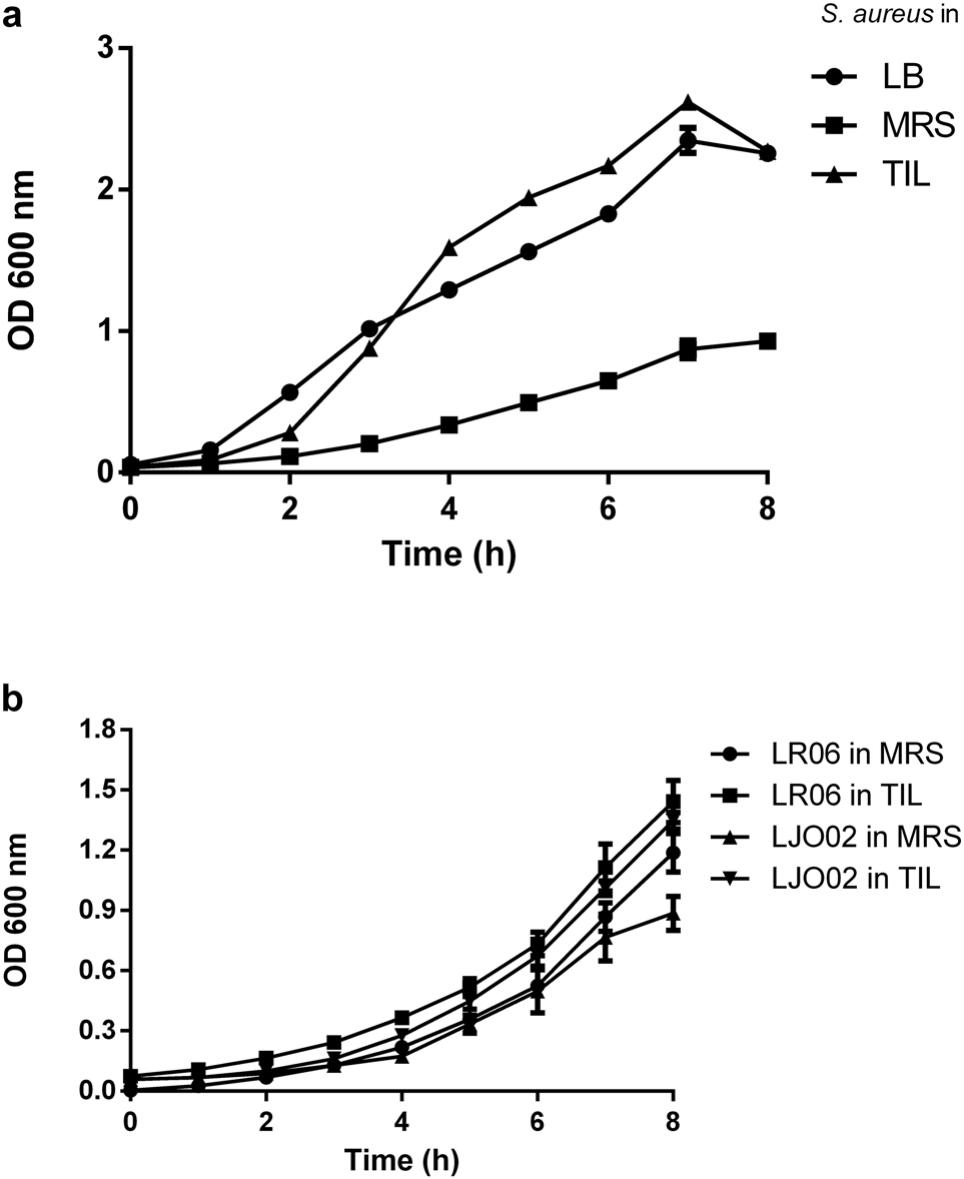
*Bacterial strain growth curves*. (**a**) *S. aureus* growth curves in LB, MRS, and TIL. (**b**) LR06 and LJO02 growth curves in MRS and TIL. Data are expressed as mean ± SD. All the experiments (n = 3) were repeated 3 times on different days. OD = optical density.

*S. aureus* was grown in the standard LB medium, and in MRS and TIL broths in which the LAB were cultured (Fig. 1a). *S. aureus* grows better in TIL, by reaching and exceeding the OD values obtained in LB, with respect to MRS, in which a reduced growth and a longer adaptation lag phase are observed.

LR06 and LJO02 strains were cultured in both MRS and TIL (Fig. 1b). Both LAB grow well in the medium containing ingredients of animal origin as well as in the animal derivative-free TIL broth. Worth of note, LJO02 growth starts slowing down after 8 h culture in MRS, while this strain remains in the exponential phase in TIL.

All the experiments (n = 3) were repeated 3 times for each strain on different days.

### Culture medium and CFS pH evaluation

Regardless of the culture media in which they are produced, CFSs acidify them, leading to pH values around 4 (LB addition does not modify these values; Table 1).

**Table 1:** pH measurement of culture media and CFSs used in the study.

	pH
LB medium	6.6
MRS medium	6.1
TIL medium	6.8
CFS from LR06 cultured in MRS	4.4
CFS from LR06 cultured in TIL	4.3
CFS from LJO02 cultured in MRS	4.2
CFS from LJO02 cultured in TIL	4.0
LB + CFS from LR06 cultured in MRS/TIL	4.4
LB + CFS from LJO02 cultured in MRS/TIL	4.3

pH values of LB, alone and plus an equal volume of each one of the CFSs produced by the LAB in MRS and TIL media. The pH of pristine MRS and TIL broths is also shown.

### pH influence on *S. aureus* growth and virulence

**Figure 2:**
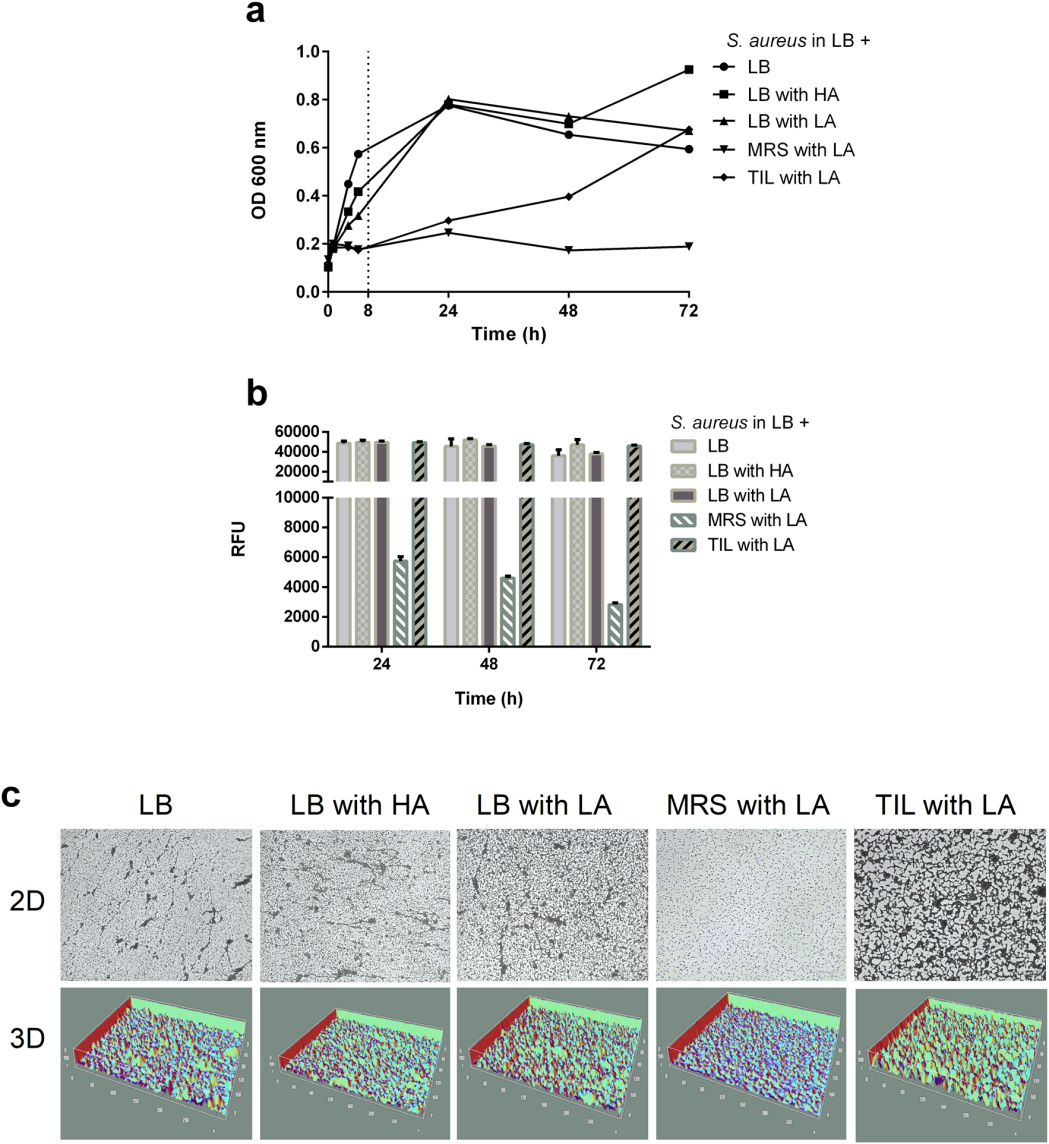
*Comparison of S. aureus growth and biofilm formation in standard and acidic media*. (**a**) Pathogen growth curves in standard LB and acidic media (pH = 4.3). Data are expressed as mean ± SD. All the experiments (n = 4) were repeated 3 times on different days. OD = optical density. HA = hydrochloric acid. LA = lactic acid. (**b**) Biofilm viability assay in standard and acidic media after 24, 48, and 72 h incubation. Data are expressed as mean ± SD of 3 independent experiments (n = 4). RFU = relative fluorescence units. (**c**) Representative 2D and 3D images of crystal violet (CV) stained *S. aureus* biofilm at 72 h grown in LB and acidic media. 2D pictures magnification: 460X. Scale bar: 100 μm.

*S. aureus* growth curves were produced in LB and in the same medium added with an equal amount of LB, MRS, and TIL after their acidification with hydrochloric or lactic acid to pH values comparable to those of the CFSs produced in MRS and TIL. The pathogen maintains its growth capacity not only in the presence of hydrochloric acid but also of lactic acid in all the tested conditions, except for acidic MRS (Fig. 2a).

*S. aureus* retains the capacity to adhere and grow as biofilm in all the conditions, as depicted by the alamarBlue assay and CV staining, except for LB plus acidic MRS, which per se seems to influence its virulence (Fig. 2b,c).

All the experiments (n = 4) were repeated 3 times for each condition on different days.

### TIL and MRS CFS activity versus *S. aureus* growth and virulence

#### Optical density evaluation

**Figure 3:**
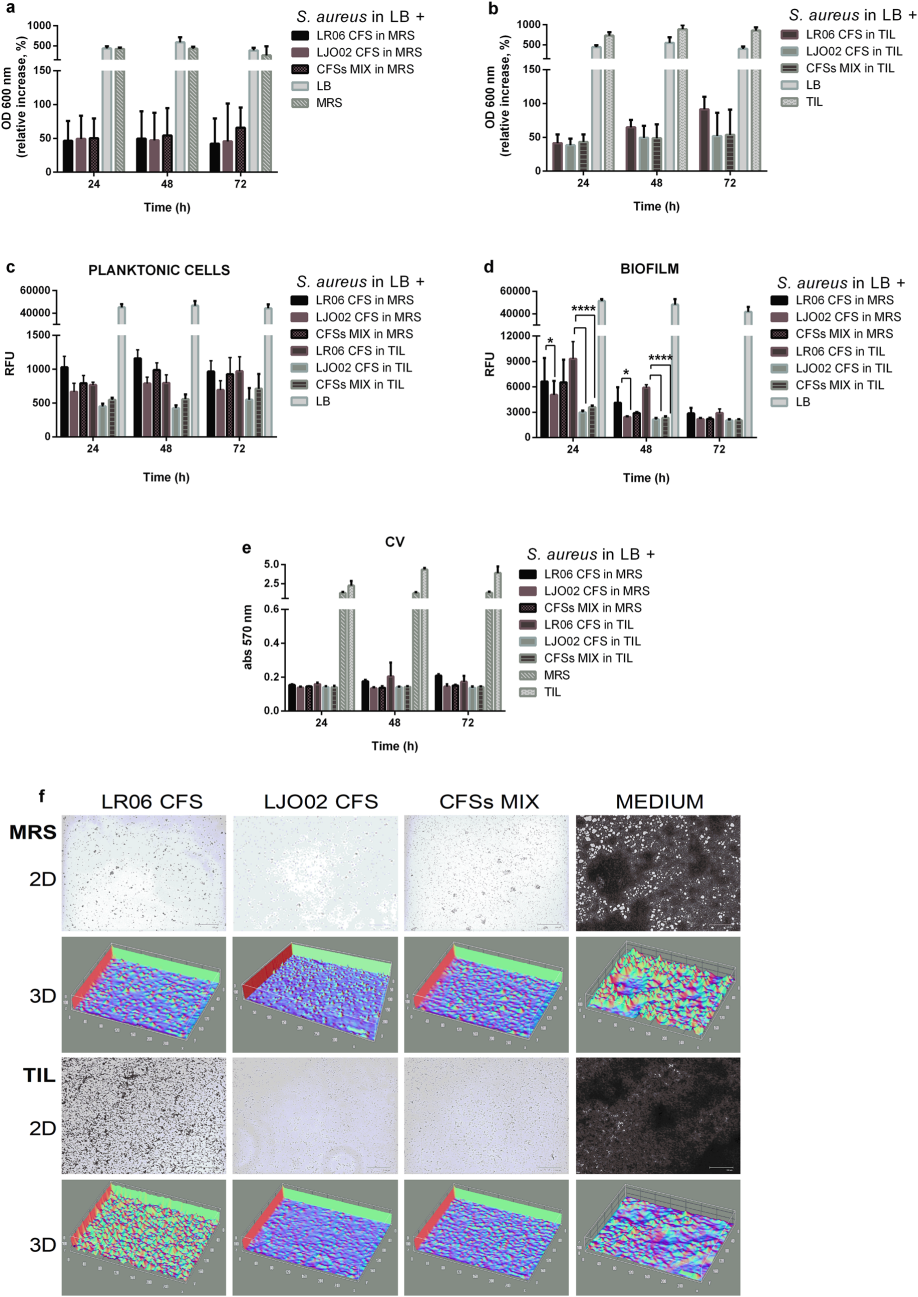
*Evaluation of the effect of single and mixed CFSs produced in MRS and TIL on S. aureus growth and biofilm formation*. *S. aureus* planktonic cells and biofilm OD_600_ after 24, 48, and 72 h of treatment with CFSs from LR06, LJO02 and their combination (MIX condition) produced in (**a**) MRS and (**b**) TIL (considering T_0_ = 100% strain viability in each experimental condition). OD = optical density. CFSs = cell-free supernatants. (**c**) Planktonic cell and (**d**) biofilm *S. aureus* viability assessment after 24, 48, and 72 h of treatment with CFSs from LR06, LJO02, and MIX conditions. Data are expressed as mean ± SD of 3 independent experiments (n = 4). All tested conditions significantly reduced both *S. aureus* planktonic cells and biofilm formation compared to the growth control in LB (*p* < 0.0001). Two-way ANOVA was applied with Tukey’s comparison test. *p < 0.05, **p < 0.01, ****p < 0.0001. RFU = relative fluorescence units. (**e**) CV-reading at 570 nm of *S. aureus* biofilm at the 3 tested time points. (**f**) Representative 2D and 3D images of CV-stained *S. aureus* biofilm after 72 h of treatment with the CFSs produced in MRS (top) and TIL (bottom). 2D pictures magnification: 460 X. Scale bar: 100 μm.

*S. aureus* OD_600_ was measured at T_0_, after 24, 48, and 72 h incubation with the CFSs produced in both MRS and TIL (OD_600_ at T_0_ corresponds to 100% viability). All CFSs strongly reduce the pathogen OD compared to the values of the bacteria grown in LB and LB plus MRS or TIL (Fig. 3a,b, respectively).

### Planktonic cell and biofilm viability

*S. aureus* planktonic cell and biofilm viability was analyzed using the alamarBlue assay. At all times and treatments tested, the metabolic activity of both forms is significantly reduced with respect to each growth control (*p* < 0.0001, two-way ANOVA; Fig. 3c,d). CFSs do not induce any statistically significant difference among the tested conditions in treated planktonic cells, regardless of the media (MRS/TIL) used for the probiotic cultures (Fig. 3c). Conversely, the most effective treatment in reducing the biofilm at 24 and 48 h is the one with the CFS produced by LJO02 in TIL, followed by the mix of the two supernatants in the same medium (both *p* < 0.0001) with respect to the CFS from LR06 in TIL, which shows the same trend, even if to a lesser extent (Fig. 3d). At 72 h, the reduced viability is still detectable, but with no differences among treatments.

#### Biofilm staining with crystal violet

The biofilm was stained with CV at the three time points selected. All the treatments display a huge effect in the reduction of biofilm formation and thickness. LJO02 CFSs and the MIX condition show a constant performance up to 72 h, while LR06 CFSs are less effective (Fig. 3e,f, and Supplementary Fig. 1S).

### Efficacy of modified LJO02 CFSs produced in TIL versus *S. aureus* growth and virulence

#### Optical density evaluation

**Figure 4:**
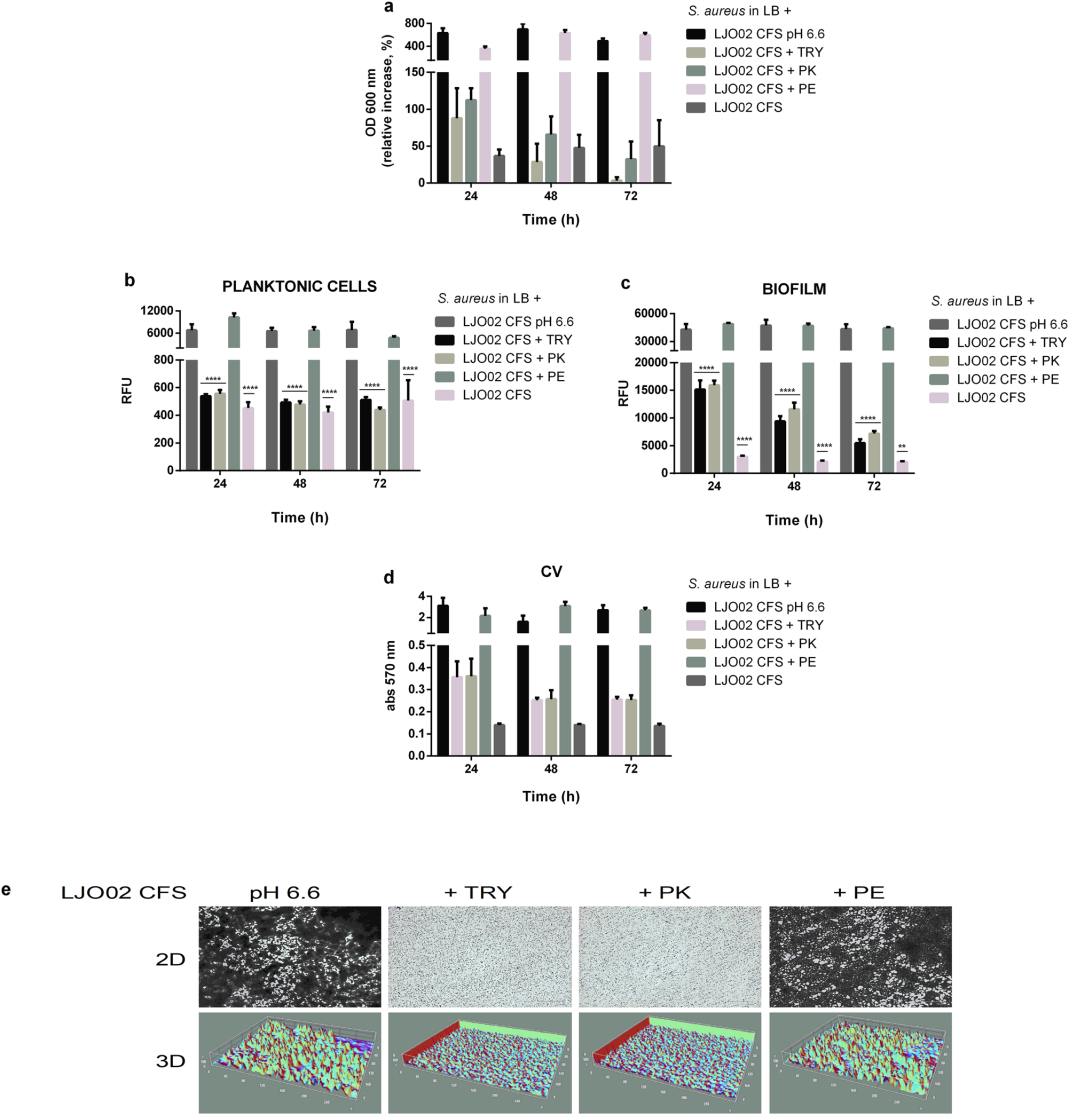
Evaluation of the effect of modified CFSs from LJO02 produced in TIL on *S. aureus* growth and biofilm formation. (**a**) *S. aureus* planktonic cell and biofilm OD_600_ after 24, 48, and 72 h of incubation with the CFSs from LJO02 in TIL at pH 6.6 or enzymatically-digested (considering T_0_ = 100% viability in each experimental condition). OD = optical density. CFS = cell-free supernatant. TRY = trypsin; PK = proteinase K; PE = pepsin. (**b**) Planktonic cell and (**c**) biofilm *S. aureus* viability assessment after 24, 48, and 72 h of incubation with modified CFSs. Data are expressed as mean ± SD of 3 independent experiments (n = 4). Trypsin and proteinase K-treated CFSs significantly reduce the planktonic cell viability compared to basification and pepsin treatment, similarly to what occurs with the untreated CFS (*p* < 0.0001). Biofilm viability is significantly reduced by trypsin and proteinase K-treated CFSs with respect to basification and pepsin at all tested times (*p* < 0.0001), but undigested CFS still shows the best performance (*p* < 0.0001 at 24 and 48 h, and *p* < 0.01 at 72 h with respect to trypsin and proteinase K-digested CFS). Two-way ANOVA was applied with Tukey’s comparison test. **p < 0.01, ****p < 0.0001. RFU = relative fluorescence units. (**d**) CV-reading at 570 nm of *S. aureus* biofilm at the 3 tested time points. (**e**) Representative 2D and 3D images of CV-stained *S. aureus* biofilm after 72 h of treatment with the modified CFSs. 2D pictures magnification: 460 X. Scale bar: 100 μm.

*S. aureus* OD_600_ was measured at T_0_, after 24, 48, and 72 h incubation with the CFS from LJO02 produced in TIL and brought at pH 6.6 or digested by 3 different endopeptidases. OD_600_ readings decrease in the presence of CFSs digested by trypsin and proteinase K with respect to those observed with CFS treated with pepsin or basified; in particular, trypsin-digested CFS OD_600_ values at 48 and 72 h exhibit lower values compared to untreated CFS, that maintains its best performance at 24 h incubation (Fig. 4a).

#### Planktonic cell and biofilm viability

The alamarBlue assay highlights that both trypsin and proteinase K-treated CFSs modify the viability of *S. aureus* planktonic cells similarly to the one induced by the original CFS at all the tested endpoints with respect to basification or pepsin treatment (*p* < 0.0001, Fig. 4b). The biofilm viability is reduced by trypsin and proteinase K compared to basified or pepsin-digested CFSs (*p* < 0.0001, Fig. 4c); the untreated CFS maintains the best performance at all the tested time points (Fig. 4c). The basification and the pepsin treatment completely revert the action induced by the natural CFS from LJO02 at all the analysed times.

#### Biofilm staining with crystal violet

Accordingly, trypsin and proteinase K treatments reduce *S. aureus* biofilm formation, even if the widest decrease is observed with the undigested CFS (Fig. 4d,e, and Supplementary Fig. 2S).

### SCFA and lactic acid content of CFSs produced in MRS and TIL

**Figure 5:**
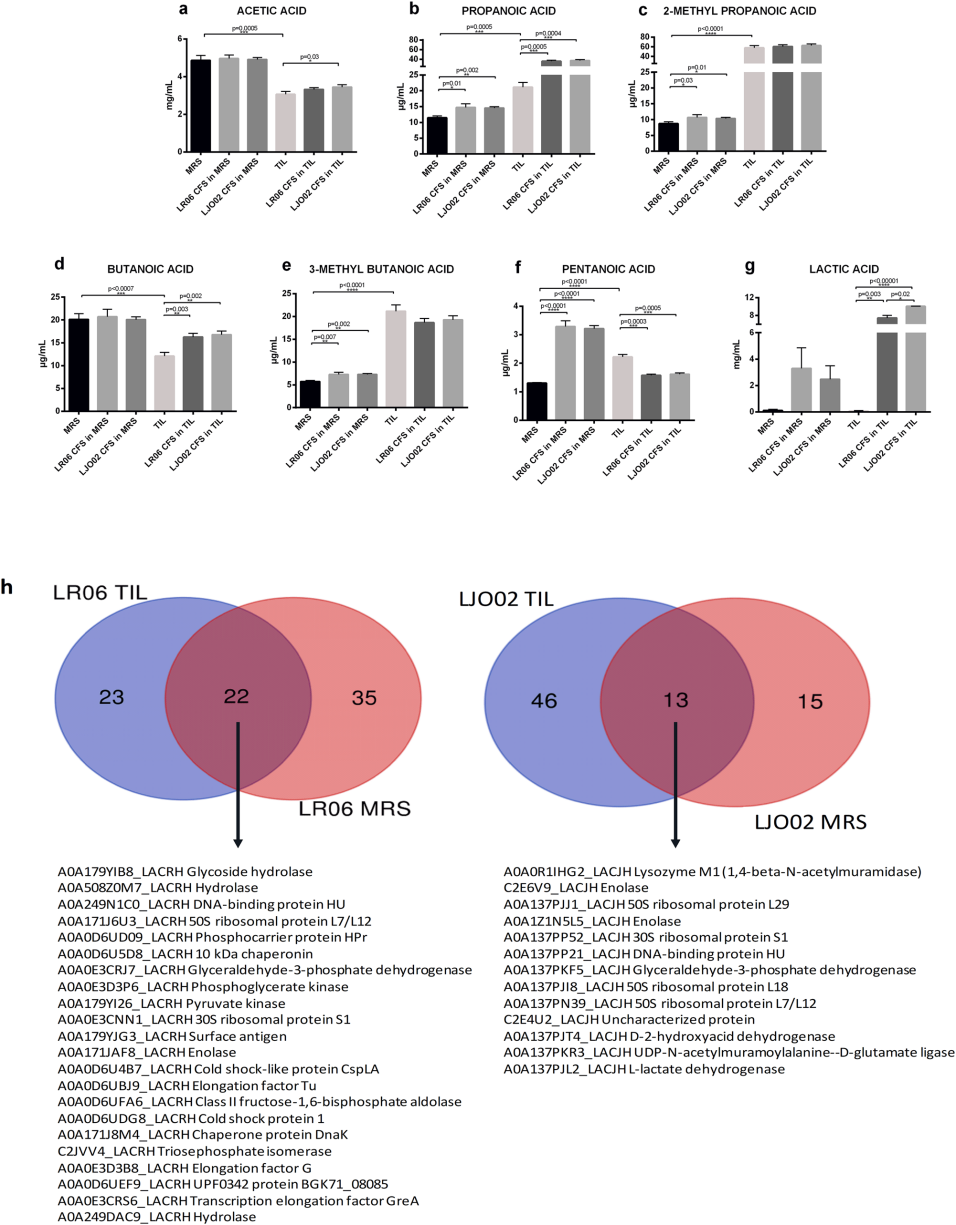
*Quantification of SCFAs and characterization of protein content in CFSs from LR06 and LJO02 cultured in MRS and TIL for 8 h*. (**a**–**g**) SCFAs amount in CFSs from LR06 and LJO02 produced in MRS and TIL. An unpaired t-test was applied. (**h**) Proteins identified in both MRS and TIL broths, and CFSs from LR06 and LJO02 cultured in both media.

The SCFA content of MRS and TIL media, together with that of the CFSs produced by LAB in MRS and TIL after an 8 h culture, was measured with gas chromatography coupled to mass spectrometry, while the lactic acid content was calculated at 340 nm based on NADH amount formation (Fig. 5). Both the basal culture broths are characterized by a strong presence of SCFAs; in particular, the acetic acid results about 100-fold more concentrated with respect to the other molecules. By comparing the basal media, MRS has higher acetic and butanoic acid levels compared to TIL, while propanoic, 2-methyl propanoic, 3-methyl butanoic and pentanoic acids are more concentrated in the animal derivative-free medium. No lactic acid is present in both the media.

Although the acetic acid is the prevalent SCFA of the media, no changes in its concentration are observed in CFSs from LAB cultivated in MRS, while only a small increase is reported in that from LJO02 in TIL (Fig. 5a). Propanoic acid concentration increases after 8 h for both LR06 and LJO02 strains cultured in MRS and TIL broths, with a more drastic increment in TIL (Fig. 5b). Two-methyl propanoic acid does not significantly increase in the CFSs produced in TIL, differently from what happens in MRS (Fig. 5c). Interestingly, butanoic acid (butyric acid) levels increase in both LR06 and LJO02 cultured in TIL (Fig. 5d). An opposite behavior is observed for 3-methyl butanoic acid, also called isovaleric acid: its amount increases in the CFSs from LR06 and LJO02 in MRS, while it reduces in TIL, although with no statistically significant differences (Fig. 5e). The same behavior is observed for pentanoic acid, namely valeric acid, which is produced by both the strains in MRS; on the other hand, significant consumption of the molecule by the LAB is observed in TIL (Fig. 5f). Finally, huge lactic acid production is detected, with the highest level in the CFS from LJO02 cultured in TIL (Fig. 5g).

### Proteins released by LAB in MRS and TIL

Untargeted proteomic analysis was performed on CFSs produced by LAB in MRS and TIL after an 8 h culture. The analysis identified a total of 80 proteins generated by LR06, 45 of which in TIL sample, and 57 in MRS. Only 22 proteins are present in both the media. Regarding LJO02, 74 proteins were identified, of which 59 in TIL, and 28 in MRS. Thirteen proteins are present in both media (Fig. 5h).

Interestingly, several enzymes with an important role in bacteria metabolism are present in the CFSs from LR06 and LJO02 cultured in both media. In particular, glycoside hydrolase, phosphoglycerate kinase, pyruvate kinase, enolase, aldolase, and isomerase are among the common proteins detected in LR06 grown in both MRS and TIL, while enolase, glyceraldehyde-3-phosphate dehydrogenase, lactate dehydrogenase, and D-glutamate ligase are present in the CFSs produced by LJO02. In addition, several ribosomal proteins are present in the CFSs from both LAB (Fig. 5h).

To better investigate the biological role of the proteins secreted by LR06 and LJO02 cultured in TIL, a Gene Ontology (GO)-functional annotation cluster analysis was performed using the DAVID software. The most represented secreted proteins by LR06 are reported in Table 2. The clustering analysis reveals enriched pathways, including protein biosynthesis, carbon metabolism, glycolysis, gluconeogenesis, microbial metabolism in diverse environments, and biosynthesis of amino acids.

**Table 2:** GO-functional annotation clusters for *L. rhamnosus* proteins.

Annotation Cluster 1	Enrichment Score: 2.45		
Category	Associated Term	Gene	*p *value
UP_KEYWORDS	Elongation factor	3	8,19E-04
GOTERM_MF_DIRECT	GO:0,003,746 ~ translation elongation factor activity	3	0,00,231
UP_KEYWORDS	Protein biosynthesis	3	0,02,357

Annotation Cluster 2	Enrichment Score: 1.81		
*Category*	*Associated Term*	*Gene*	*p-value*
KEGG_PATHWAY	lrh01200: Carbon metabolism	5	3,96E-04
KEGG_PATHWAY	lrh00010: Glycolysis / Gluconeogenesis	4	0,00,151
GOTERM_BP_DIRECT	GO:0,006,096 ~ glycolytic process	3	0,00,527
KEGG_PATHWAY	lrh01120: Microbial metabolism in diverse environments	5	0,00,636
KEGG_PATHWAY	lrh01230: Biosynthesis of amino acids	4	0,0171
KEGG_PATHWAY	lrh01130: Biosynthesis of antibiotics	4	0,0382

The following clusters, resulting from the DAVID-GO Functional Annotation Clustering, represent the secreted proteins of LR06 cultured in TIL.

The most produced proteins by LJO02 are reported in Table 3. The clustering analysis reveals enriched pathways including ribosome and protein translation, gluconeogenesis, glycolysis, biosynthesis of secondary metabolites, and carbon metabolism.

**Table 3:** GO-Functional Annotation Clusters for *L. johnsonii* proteins.

Annotation cluster 1	Enrichment Score: 4.78		
Category	Associated term	Gene	*p* Value
UP_KEYWORDS	Ribosomal protein	8	8,12E-07
UP_KEYWORDS	Ribonucleoprotein	8	9,20E-07
GOTERM_MF_DIRECT	GO:0,003,735 ~ structural constituent of ribosome	8	5,56E-06
GOTERM_CC_DIRECT	GO:0,005,840 ~ ribosome	7	2,42E-05
GOTERM_BP_DIRECT	GO:0,006,412 ~ translation	8	2,29E-04
KEGG_PATHWAY	ljo03010: Ribosome	8	8,01E-04

Annotation cluster 2	Enrichment Score: 1.86		
Category	Associated term	Gene	*p* Value
UP_KEYWORDS	Gluconeogenesis	3	6,58E-04
GOTERM_BP_DIRECT	GO:0,006,096 ~ glycolytic process	4	8,58E-04
KEGG_PATHWAY	ljo00010: Glycolysis / Gluconeogenesis	6	9,35E-04
KEGG_PATHWAY	ljo01130: Biosynthesis of antibiotics	7	0,00,376
UP_KEYWORDS	Glycolysis	3	0,00,382
GOTERM_BP_DIRECT	GO:0,006,094 ~ gluconeogenesis	3	0,00,753
KEGG_PATHWAY	ljo01110: Biosynthesis of secondary metabolites	7	0,00,883
KEGG_PATHWAY	ljo01120: Microbial metabolism in diverse environments	6	0,00,997
KEGG_PATHWAY	ljo01200: Carbon metabolism	5	0,0200

The following clusters, resulting from the DAVID-GO Functional Annotation Clustering, represent the secreted proteins of LJO02 cultured in TIL.

## Discussion

In the present work, we analyzed the response of a MRSA strain (*S. aureus* ATCC 43300), an example of an opportunistic human pathogen belonging to the ESKAPE group, to the treatment with single, mixed, and modified supernatants produced by two selected LAB (*L. rhamnosus* LR06 DSM 21981 and *L. johnsonii* LJO02 DSM 33828), both grown in an animal derivative-based commercial MRS medium and a novel animal derivative-free TIL broth.

The final aims of this research were to find the best LAB candidate and growing condition to produce CFSs able to limit *S. aureus* growth and virulence, and possibly identify the more determinant key SCFAs and proteins in the CFSs that could counteract the pathogen viability, metabolism, and biofilm formation.

Therefore, we assessed the different activity of six single and mixed CFSs, produced by the two LAB both grown in MRS and TIL, by using combined culturomic, pH shift, enzymatic, gas chromatographic/mass spectrometric, proteomic and metabolic approaches.

Before incubating the MRSA with the CFSs, we evaluated its growth behavior and biofilm formation in LB, MRS, and TIL media, before and after acidification. *S. aureus* adapts to almost all tested broths but exhibits a reduced growth in MRS, which is abolished when lactic acid is added. The different response was partially explained by Zhou et al*.*, who documented how *S. aureus* is more resistant to inorganic strong acids, such as the hydrochloric, and less toward weak organic ones, such as acetic or lactic acids, because of their incomplete dissociation in water^27^. Undissociated weak acids enter the bacteria and release protons, inducing intracellular acidification that leads to bacteria viability decrease.

The two homofermentative LAB produce high amounts of lactic acid from the enzymatic fermentation of sugars and demonstrate a similar growth capacity in the two different culture media, except for LJO02 in MRS, whose log phase starts slowing down around 8 h culture. This is likely due to the vegetal extract, which enhances and extends its growth, as observed for other bacteria cultured in media with components of such origin^28^.

As a next step, we assessed the response of *S. aureus* planktonic cells and biofilm to the treatment with the CFSs produced in MRS and TIL, alone and in combination. In our study model, a general strong OD_600_ reduction is observed with all the treatments compared to the untreated controls at all the tested time points. Each treatment strongly reduces the metabolic activity of both *S. aureus* forms with respect to the growth control. In particular, the CFS from LJO02 cultured in TIL, with respect to MRS, is the most effective in reducing the biofilm expression. The effect of the CFS from LJO02 produced in TIL on biofilm viability at 24 and 48 h is similar to what is obtained in the MIX condition; the CFS from LR06 follows the same trend, albeit to a lesser extent. The absence of differences among treatments at 72 h could be due to the slower action of the CFS from LR06, and the reduction over time of LJO02 CFS efficacy. TIL shows to be the more suitable substrate for the maintenance of LAB viability and metabolism, thus favouring *S. aureus* virulence containment by the CFSs. Moreover, the pathogen viability reduction is not observed by the simple media acidification with lactic acid, except for MRS. This result reinforces the data on the efficacy of the CFSs demonstrated by several authors^20,29,30^. To support this evidence, Luo et al*.* underlined that also *S. aureus* produces lactic acid that not only allows its survival but also contributes to its aggregation^31^. Therefore, the inhibitory effect on pathogen growth and biofilm formation could be likely due to CFS factors other than the lactic acid.

Importantly, CV staining reveals that in all the treatment conditions *S. aureus* seems no more able to constitute a biofilm with respect to the control, with only a few aggregated cells adhering to the surface, as previously reported by Melo et al*.*^32^.

To better assess if the pH alone could affect the anti-bacterial properties of the CFS from LJO02 in TIL, we basified it with NaOH to the pH value of 6.6, the same as pristine LB. Moreover, to achieve information about the activity of the proteins contained in the CFS from LJO02 in TIL, we treated it with trypsin, proteinase K, or pepsin. Modified CFSs partially revert *S. aureus* OD_600_ reduction promoted by the untreated CFS. The reduction of planktonic cell viability obtained with the CFS treated with trypsin and proteinase K is comparable to those obtained with undigested CFS, which maintains the best performance in reducing biofilm metabolic activity at all tested times. This result underlines the need for the peptide bonds to be intact when basic amino acids are involved (such as arginine and lysine) compared to the aromatic ones (as tyrosine, tryptophan, and phenylalanine) to maintain CFS activity^33^.

To obtain a detailed molecular understanding of the CFSs produced by the LAB in MRS and TIL, we carried out SCFAs, lactic acid, and proteomic qualitative and quantitative comparisons. By using a high-resolution mass spectrometry and gas chromatography combined approach, we demonstrated that MRS and TIL media are free from lactic acid, while they exhibit a high and specific SCFA content, with an acidic composition that differs by type and quantity. Despite LR06 and LJO02 show the same growth curve trend in the two broths, the different media composition and their metabolic activity determine the diverse influence of their CFSs on the ability of the MRSA to grow and form biofilm. The TIL medium strongly stimulates the increase of propanoic, butanoic, and lactic acids in the CFSs from both the LAB, but mainly by LJO02. Propanoic acid and its other form with 2-methyl substituent are recognized for their ability to inhibit the growth of molds and possess well-documented antimicrobial characteristics against a broad spectrum of Gram-positive and Gram-negative bacteria. They also induce bacterial membrane permeabilization, depolarization, damage, and final disruption, thus acting as potential prodrugs^34^.

Kennedy et al. demonstrated the antibacterial property of butanoic acid, also known as butyric acid, and its 2-branched fatty acid towards a broad-spectrum of MDR bacteria^35^. Indeed, these acids are FDA-approved safe additives (https://www.accessdata.fda.gov/scripts/cdrh/cfdocs/cfcfr/cfrsearch.cfm?fr=582.60), also recognized as “green chemistry” disinfectants^36^. The isovaleric (3-methyl butanoic) acid is normally produced and released by *S. aureus*^37^; it is more present in TIL with respect to MRS and decreases in the two CFSs produced in TIL in a similar but not statistically significant way. LAB produce lactic acid in both the media, but the animal derivative-free components of TIL enhance their metabolic activity, in particular the production of this organic acid by LJO02. This carboxylic acid, also recognized as 2-hydroxypropanoic acid, is the main product of sugar metabolism and may inhibit important pathogens, targeting their cell wall and cytoplasmic membrane. Moreover, it induces specific metabolic properties, such as replication and protein synthesis, leading to pathogen inactivation or even death^38^. It also creates an unfavorable microenvironment by completely inhibiting the growth of bacteria such as *Salmonella* spp*., Escherichia coli,* or *Listeria monocytogenes,* without affecting host epithelial cells^39^.

The proteomic analysis retrieved 22 identical proteins in the CFSs produced by LR06 in both the media, with 57 produced in MRS and 45 in TIL. The CFSs produced by LJO02 contain a total of 74 proteins: 13 in common between the two media, 28 produced in MRS, and 59 in TIL. Interestingly, we identified 11 common proteins between the CFSs produced in TIL by LR06 and LJO02: DNA-binding protein HU, triosephosphate isomerase, phosphoglycerate kinase, elongation factor Ts, 2,3-bisphosphoglycerate-dependent phosphoglycerate mutase, ABC transporter substrate-binding protein, hydrolase, 30S ribosomal protein S1, enolase, 50S ribosomal protein L7/L12, and glyceraldehyde-3-phosphate dehydrogenase. Among these proteins, there are several enzymes of the central metabolism involved, in their phosphorylated forms, in the life of *S. aureus*^40^. Cells use signal-transducing pathways through a receptor that receives a signal that is related to other components inside the cell, and phosphorylation is one of the most used events to transmit signals. This suggests that glycolytic enzymes produced in TIL could have a potential role in the inhibition of *S. aureus.* Moreover, Kang et al. performed a proteomic analysis on the supernatants produced by a *L. salivarius* strain, identifying a total of 5 secreted proteins, including a Lys M-containing peptidoglycan binding protein and a protein peptidase M23B, suggesting their potential efficacy against *S. aureus* biofilm^30^.

Despite the importance of our discoveries, further experiments are needed to investigate the possible signaling role of these proteins against *S. aureus* pathogenicity and virulence. We will examine the phosphorylation status of the proteins contained in the CFSs produced in TIL. Since the changes in their charge, responsible for conformational variations, substantially modify their binding to ligands and their differential activity, we will conduct expression and functional phospho-proteomic analysis by mass spectroscopy approaches. The proteins express their activity via complex signaling pathways which intersect each other, thus forming signal networks that determine biological responses. Identifying the phosphorylated sites will be essential to clarify and understand different phenomena and signal transduction pathways which will allow us to fully comprehend the roles that these proteins may play. We will make an in-depth qualitative comparison of the protein content, with the final aim to attribute to each component novel peculiar functions. Meanwhile, we will conduct experiments in which CFSs will be repeatedly added to the MRSA pathogen during the pre-established culture media renewals. This last aspect will help clarify if the CFS activity is limited in time and/or reversible, and if their summation effect is time/dose-dependent, steadily reduces the virulence, and restores the commensal phenotype of the MRSA.

## Conclusion

A deeper knowledge of the CFS potential will open new therapeutic frontiers, which will be achieved by eventually combining their use with conventional approaches. Thus, they could reduce the progression and severity of the typical symptoms of MRSA and other ESKAPE pathogen infections and facilitate the development of targeted pharmaceutical intervention. The direct antagonism obtained with non-pathogenic probiotic bacteria, or their postbiotic products, could have direct application in human prevention, bacteriotherapy, and bioremediation of hospital equipment surfaces.

This is the first investigation showing that an animal derivative-free broth can stimulate, to a greater extent than an animal derivative-based medium, the production of CFSs able to counteract opportunistic *S. aureus* growth and virulence, possibly through specific regulators.

## Supplementary Information

Supplementary Information.

## Data Availability

The datasets generated and/or analysed during the current study are available via ProteomeXchange with the identifier PXD030916. Supplementary Figs. 1S and 2S are available in the Supplementary Information file of this paper.
