# Supplementary material for: An animal derivative-free medium enhances Lactobacillus johnsonii LJO02 supernatant selective efficacy against the methicillin (oxacillin)-resistant Staphylococcus aureus virulence through key-metabolites
Source: Sci Rep. 2022 May 23;12:8666. doi: 10.1038/s41598-022-12718-z (PMC9126979; doi:10.1038/s41598-022-12718-z)
Supplement: Supplementary file 1 — Supplementary Information. [file 41598_2022_12718_MOESM1_ESM.pdf]

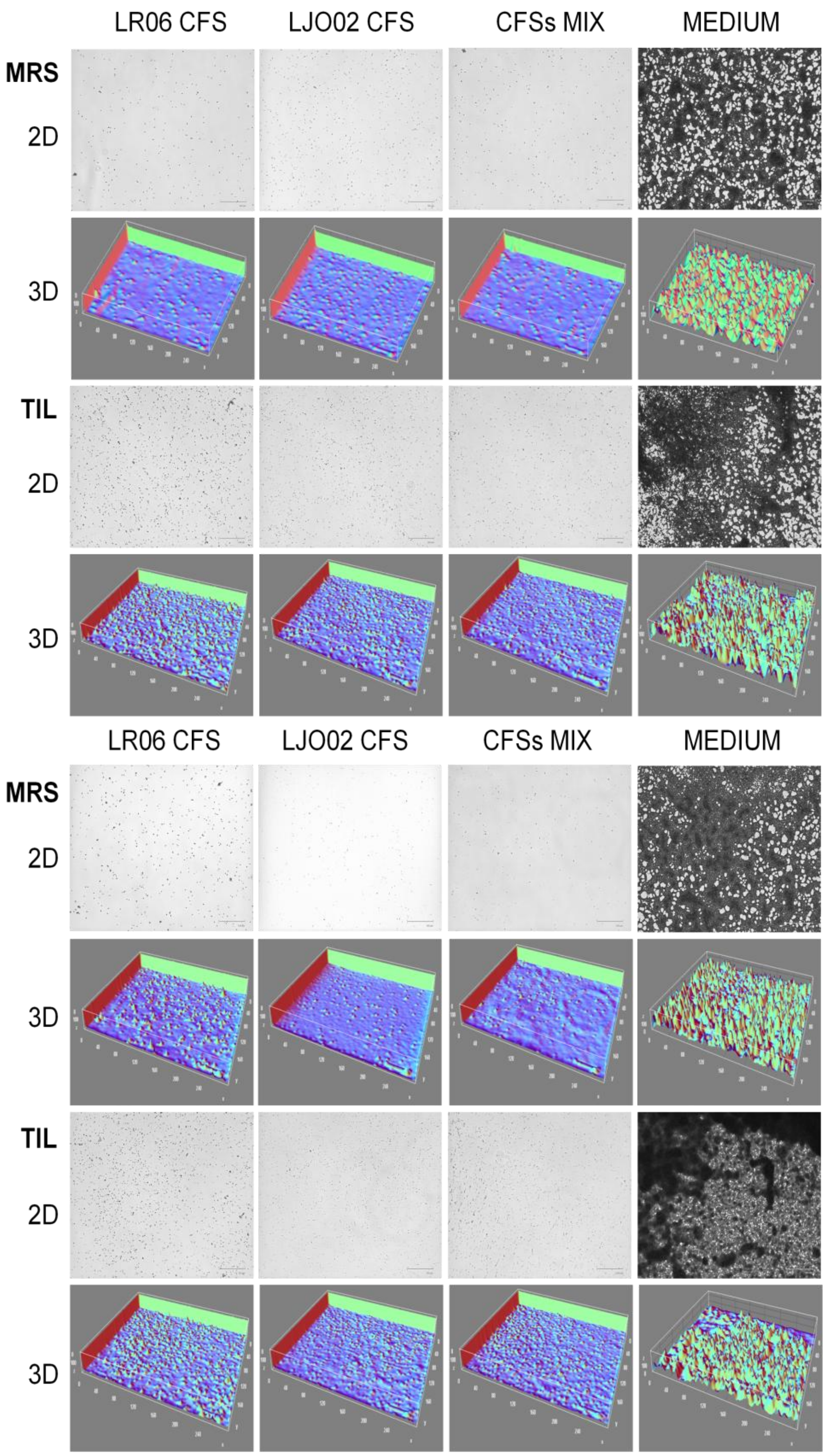

Supplementary Figure 1S. CV-stained *S. aureus* biofilm after treatment with CFSs in MRS and TIL. Representative 2D and 3D images of CV-stained *S. aureus* biofilm after 24 h (upper panel) and 48 h (lower panel) of incubation with the CFSs produced in MRS and TIL. 2D pictures magnification: 460 X. Scale bar: 100 µm.

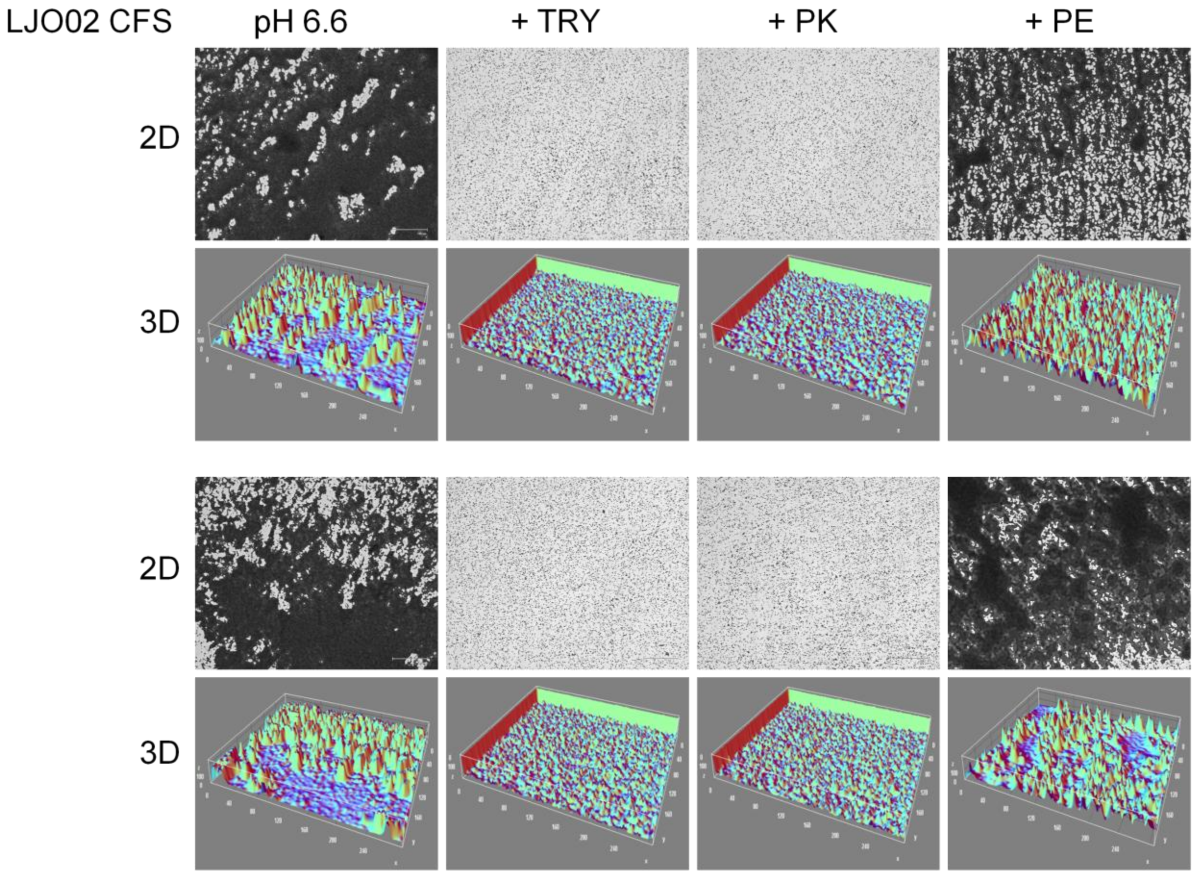

Supplementary Figure 2S. CV-stained *S. aureus* biofilm after treatment with LJO02 CFSs produced in TIL with adjusted pH or treated with proteolytic enzymes. Representative 2D and 3D images of CV-stained *S. aureus* biofilm after 24 h (upper panel) and 48 h (lower panel) of incubation with the modified CFSs. 2D picture magnification: 460 X. Scale bar: 100 µm.
